# Exploring the relationship between intestinal microbiota and immune checkpoint inhibitors in the treatment of non-small cell lung cancer: insights from the “lung and large intestine stand in exterior-interior relationship” theory

**DOI:** 10.3389/fcimb.2024.1341032

**Published:** 2024-02-09

**Authors:** Luwei Li, Hongmei Zhong, Yajie Wang, Zongying Pan, Shumei Xu, Shuai Li, Guilin Zeng, Weiwei Zhang, Jie Li, Lang He

**Affiliations:** ^1^ Chengdu University of Traditional Chinese Medicine, Chengdu, Sichuan, China; ^2^ Cancer Prevention and Treatment Institute of Chengdu, Department of Oncology, Chengdu Fifth People’s Hospital (The Second Clinical Medical College), Affiliated Fifth People's Hospital of Chengdu University of Traditional Chinese Medicine, Chengdu, Sichuan, China; ^3^ Center for Medicine Research and Translation, Chengdu Fifth People’s Hospital, Chengdu, China

**Keywords:** biomarkers of intestinal flora, immunotherapy, non-small cell lung cancer, TCM syndrome type, immune microenvironment, lung and large intestine

## Abstract

**Objective:**

This study is aim to discern the Traditional Chinese Medicine (TCM) syndrome classifications relevant to immunotherapy sensitive in non-small cell lung cancer (NSCLC) patients, and to delineate intestinal microbiota biomarkers and impact that wield influence over the efficacy of NSCLC immunotherapy, grounded in the TCM theory of “lung and large intestine stand in exterior-interior relationship.”

**Methods:**

The study cohort consisted of patients with advanced NSCLC who received treatment at the Oncology Department of Chengdu Fifth People’s Hospital. These patients were categorized into distinct TCM syndrome types and subsequently administered immune checkpoint inhibitors (ICIs), specifically PD-1 inhibitors. Stool specimens were collected from patients both prior to and following treatment. To scrutinize the differences in microbial gene sequences and species of the intestinal microbiota, 16S rRNA amplicon sequencing technology was employed. Additionally, peripheral blood samples were collected, and the analysis encompassed the assessment of T lymphocyte subsets and myeloid suppressor cell subsets via flow cytometry. Subsequently, alterations in the immune microenvironment pre- and post-treatment were thoroughly analyzed.

**Results:**

The predominant clinical manifestations of advanced NSCLC patients encompassed spleen-lung Qi deficiency syndrome and Qi-Yin deficiency syndrome. Notably, the latter exhibited enhanced responsiveness to ICIs with a discernible amelioration of the immune microenvironment. Following ICIs treatment, significant variations in microbial abundance were identified among the three strains: Clostridia, Lachnospiraceae, and Lachnospirales, with a mutual dependency relationship. In the subset of patients manifesting positive PD-L1 expression and enduring therapeutic benefits, the study recorded marked increases in the ratios of CD3^+^%, CD4^+^%, and CD4^+^/CD8^+^ within the T lymphocyte subsets. Conversely, reductions were observed in the ratios of CD8%, Treg/CD4^+^, M-MDSC/MDSC, and G-MDSC/MDSC.

**Conclusion:**

The strains Clostridia, Lachnospiraceae, and Lachnospirales emerge as potential biomarkers denoting the composition of the intestinal microbiota in the NSCLC therapy. The immunotherapy efficacy of ICIs markedly accentuates in patients displaying durable treatment benefits and those expressing positive PD-L1.

## 1 Introduction

Statistics reveal that lung cancer accounts for one in every five cancer-related fatalities, approximately totaling 1.8 million cases. This malignancy, characterized by its deleterious impact, poses a significant public health challenge (Sung et al., 2021). Although the exact etiological factors responsible for the development of lung cancer remain elusive, established contributors to its escalating incidence encompass smoking, air pollution, occupational exposures, and genetic predisposition (Chen et al., 2010; Bade and Dela Cruz, 2020). Lung cancer, delineated by its pathophysiological attributes, can be categorized as a malady characterized by deficiency and hyperplasia. Within the purview of traditional Chinese medicine (TCM), this affliction is perceived as an accumulation of the deficiency of vital “Qi,” a concept encompassing the vitality of life forces. This typology of lung cancer principally stratifies into the realms of Qi deficiency, Yin deficiency, and Qi-Yin deficiency, among other subtypes. It comprises the two primary histological subtypes, non-small cell lung cancer (NSCLC) and small cell lung cancer (SCLC), with NSCLC constituting the prevailing manifestation (Herbst et al., 2018; Duma et al., 2019). An insightful retrospective study, encompassing 180 cases of advanced NSCLC, unveiled a prevailing presence of Qi deficiency within lung cancer patients. Remarkably, the predominant syndrome observed among patients undergoing initial treatment was the Qi deficiency syndrome, while those in advanced stages primarily exhibited a combination of Qi deficiency and Yin deficiency (Meng et al., 2014). Furthermore, Han et al. conducted an extensive investigation involving 861 patients, elucidating that clinical stages of NSCLC were prominently characterized by lung Qi deficiency and lung Yin deficiency. Notably, the Qi-Yin deficiency syndrome predominated among patients in stages III and IV (Han et al., 2016). Consequently, the central therapeutic paradigm in the context of lung cancer, as posited in TCM, revolves around the concept of “Fuzheng Quxie.” This modality of treatment aims to bolster the body’s immunity, effectively expelling pathogenic influences.

It represents an efficacious strategy for mitigating immune evasion within advanced NSCLC by modulating tumor immune responses, enhancing immune recognition, and thereby augmenting tumor immune eradication through immunotherapy (Katayama et al., 2019). Among these approaches, immune checkpoint manipulation has emerged as the principal avenue in the realm of tumor immunotherapy (Zheng et al., 2021; Zhou et al., 2021). Concomitantly, traditional Chinese medicine also assumes an antineoplastic role through its capacity to regulate immune functions and enhance immune efficacy (Yang and Lin, 2022).For instance, ginsenosides effectively hinder the migration of lung cancer cells by facilitating the conversion of M2 macrophages to the M1 subtype through G-Rh2 (Li et al., 2018)..Yangyin Fuzheng Decoction has the capacity to modulate the expression of pro-apoptotic proteins, such as p53 and Bax, while inhibiting the expression of the anti-apoptotic protein Bcl-2 (Wei et al., 2018).Consequently, both Immune Checkpoint Inhibitors (ICIs) and traditional Chinese medicine contribute to an anti-tumor effect by bolstering the body’s immune response.

It is noteworthy that perturbations in the gut microbiota composition have been linked with various malignancies (Matson et al., 2018; Santoni et al., 2018), and recent studies have underscored a correlation between human gut microbiota and the etiology and progression of NSCLC. The dynamic interplay of these microbial populations significantly influences diverse pathways, including those associated with metabolism, inflammation, and immunity (Zhuang et al., 2019; Zheng et al., 2020; Lu et al., 2021; Zhao et al., 2021). Moreover, a growing body of evidence attests to the capacity of intestinal microbes and their metabolites to impact the efficacy of immunotherapy in NSCLC. Intervening in the intestinal bacteria can improve the immunotherapeutic response rate in NSCLC by influencing or reconfiguring the immune microenvironment (Zheng et al., 2020). However, the precise mechanisms through which intestinal microbiota exert their influence on immunotherapy remain to be elucidated, and the specific subtypes of NSCLC amenable to immune checkpoint therapy require further comprehensive exploration (Ni et al., 2021).

TCM theory emphasizes the concept of holistic health, considering individuals as integrated entities. On one front, “Fuzheng” therapy focuses on restoring the balance of intestinal microecology and enhancing immunity, thereby fortifying the body’s ability to resist the intrusion of malignancies or the forces of “pathogenic Qi.” According to TCM principles, fostering robust “righteous Qi” within an individual acts as a protective shield against malevolent influences. On the other hand, the disruption of intestinal microecology equilibrium can be construed as a state where opportunistic pathogenic microorganisms, representing the forces of “pathogenic Qi,” overpower beneficial bacteria indicative of “righteous Qi.”The theory known as the “lung and large intestine stand in exterior-interior relationship,” originally documented in the Neijing, as undergone successive refinements by subsequent generations of medical practitioners. It has gradually evolved into a foundational framework guiding the diagnosis and treatment of pulmonary and intestinal maladies, elucidating the profound interconnection between these two organ systems from both physiological and pathological standpoints. Modern research has further substantiated this close association between the lung and large intestine through the lenses of meridians, zang-fu organs, and exterior-interior relationships. Notably, empirical data indicate that approximately 68.7% of patients afflicted with chronic obstructive pulmonary disease manifest concurrent symptoms of constipation. Furthermore, the application of the “Tongfu Lichang” approach, aimed at ameliorating constipation, has demonstrated a notable capacity to concurrently alleviate pulmonary symptoms (Patel and Kurzrock, 2015). Therefore, certain scholars have posited that the “lung-intestinal correlation” may not stem directly from a fixed anatomical structure but rather entails a dynamic process governed by multifaceted factors (Wang et al., 2020). Moreover, grounded in the concept of the “gut-lung axis” (Keely et al., 2012; Dumas et al., 2018; Zhang et al., 2020), it has been established that the mucosal interfaces of the lung and intestine exhibit a closely intertwined relationship, encompassing both immunological interactions and pathological phenomena. Evidence underscores that microbial populations residing within the gastrointestinal tract can directly migrate to the lung, while chronic pulmonary ailments can precipitate imbalances in intestinal microecology and alterations in the bacterial community composition. By inhibiting intestinal bacterial propagation and thwarting bacterial translocation, effective intervention for lung diseases can be achieved (Jin et al., 2019). Antecedent investigations have also identified reductions in specific bacterial taxa, such as Votella praevoidi and Spirochaeta Rachnoi, in lung cancer patients (Liu et al., 2019; Zhang et al., 2019; Qin et al., 2022). These findings lend empirical validation to the TCM tenet of “lung and large intestine stand in exterior-interior relationship,” particularly through the prism of gut microbiota. In light of these considerations, this study endeavors to elucidate the “lung and large intestine stand in exterior-interior relationship” by exploring the role of intestinal microbiota in the context of immunotherapy for NSCLC. By synergistically integrating TCM principles with contemporary medical practice, this research seeks to offer novel insights into the underlying mechanisms governing TCM-based approaches to NSCLC treatment.

Building upon the aforementioned theoretical foundations, this study, in its initial phase, undertook the identification of TCM syndrome types within the realm of NSCLC that exhibit heightened sensitivity to immunotherapy. Subsequently, it embarked on the exploration and selection of intestinal microbiota biomarkers exerting a discernible impact on the efficacy of immunotherapy in the context of NSCLC. These biomarkers were derived from multifaceted assessments, encompassing levels of intestinal microorganisms, the composition of peripheral blood T lymphocytes, and subsets of myeloid suppressor cells, all evaluated prior to and following treatment with PD-1 inhibitors. Furthermore, the intricate interplay of intestinal microorganisms was rigorously investigated. Finally, this research delved into the profound influence of the immune microenvironment on therapeutic outcomes among NSCLC patients presenting distinct TCM syndromes. The findings underscored that, within the cohort of advanced NSCLC patients, the predominant clinical manifestations primarily manifested as spleen-lung Qi deficiency syndrome and Qi-Yin deficiency syndrome. Notably, among the myriad of identified biomarkers, specific taxa, such as Clostridia, Trichospirillales, and Trichospirillaceae, emerged as potential candidates for the treatment of NSCLC grounded in the tenet of the “lung and large intestine stand in exterior-interior relationship” theory. Moreover, the investigation illuminated that immune checkpoint inhibitors (ICIs) exhibited more pronounced efficacy within the subgroup of patients demonstrating enduring therapeutic benefits and those displaying positive PD-L1 expression. These beneficiaries of immunotherapy also exhibited a more discernible trend toward enhancement in the immune microenvironment.

## 2 Materials and methods

### 2.1 Patient

**Figure 1:**
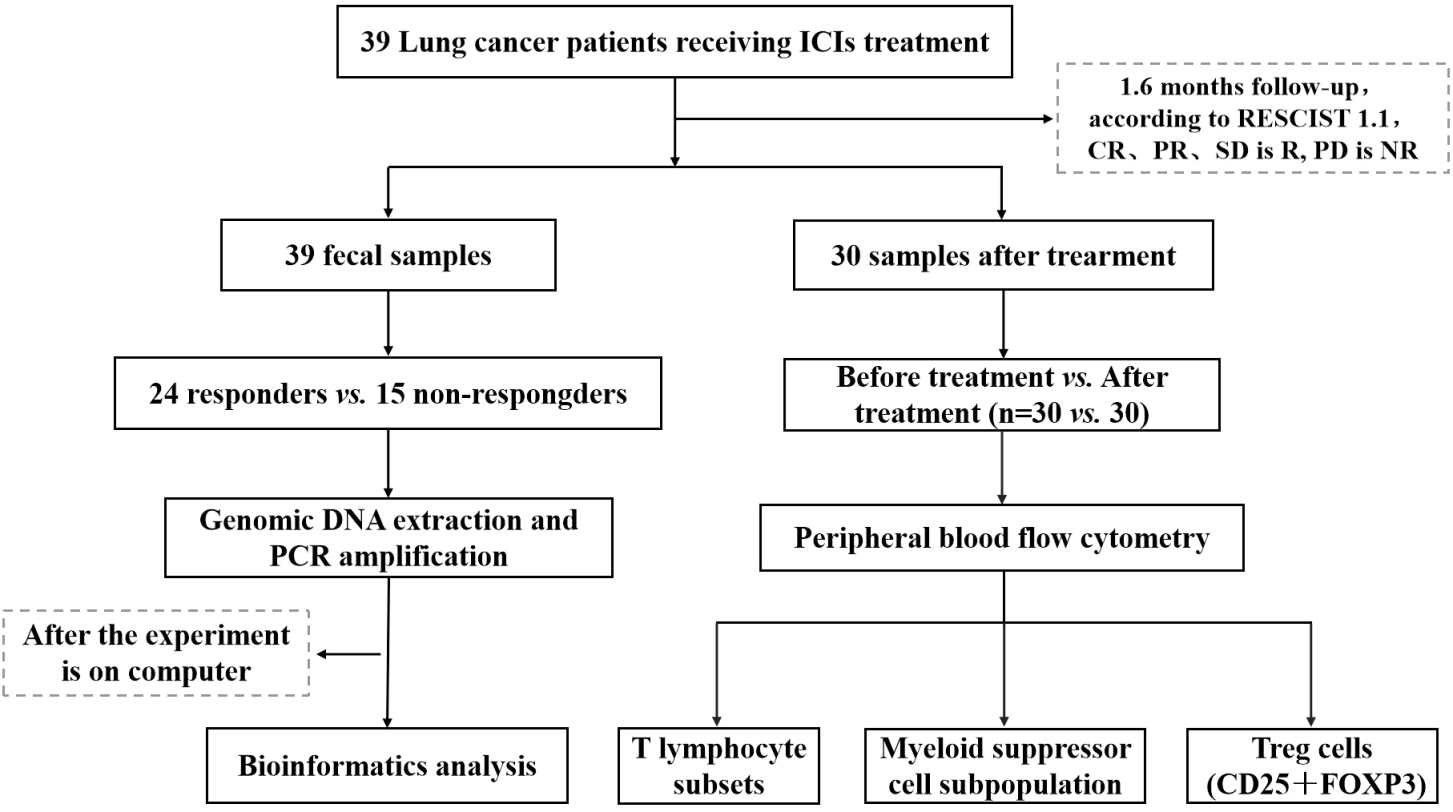
Schematic diagram of the overall process.

This study focused on a cohort of advanced NSCLC patients who sought medical care at the Oncology Department of Chengdu Fifth People’s Hospital during the period spanning December 2020 to June 2021, A total of 39 patients were enrolled by the central limit theorem. These patients were specifically prescribed PD-1 inhibitors as part of their therapeutic regimen. Comprehensive clinical data pertaining to these patients were meticulously documented, and a rigorous follow-up protocol was established, spanning until March 2022. Throughout this follow-up duration, the patients diligently adhered to their prescribed ICIs regimen, persisting within the treatment course unless confronted with disease progression, the onset of severe adverse reactions necessitating therapeutic modification or cessation, or the unfortunate event of mortality. All patients voluntarily provided informed consent for their participation in this study, and the research protocol and its ethical considerations received the requisite approval from the Ethics Committee of Chengdu Fifth People’s Hospital (Lun Review 2021-019 (Section)-01) (**Figure 1**).

The inclusion criteria were: (1) age ≥18 years; (2) anticipated life expectancy ≥ 12 weeks; (3) Eastern Cooperative Oncology Group (ECOG) score ranging from 0 to 2; (4) diagnosis of NSCLC staged as IIIB to IV, ineligibility for surgical intervention, and previous receipt of either first- or second-line systemic treatments; (5) presence of at least one objectively quantifiable tumor lesion, adhering to the criteria delineated in Immune Response Evaluation Criteria in Solid Tumors (iRECIST) version 1.1; (6) negative outcomes in driver gene testing for mutations, including KRAS, EGFR, ALK, BRAF, ROS1, etc.; (7) absence of any prior exposure to ICIs.

The exclusion criteria were: (1) individuals with established, medically documented digestive or other conditions recognized for their influence on gut microbial composition; (2) history of recent antibiotic utilization, including antifungal agents, probiotics, prebiotics, and hormonal medications, within the 1-month period preceding enrollment; (3) patients manifesting mental health disorders and cognitive impairments who have previously been subjected to pharmacological agents acknowledged for their significant perturbation of intestinal microbiota within the initial six months of the enrollment period, were deemed ineligible for participation.

### 2.2 Observation indicators and grouping

Clinical assessments encompassed a comprehensive array of parameters, including demographic variables (sex, age), pathological classification, TNM staging, ECOG score, smoking history, results of driver gene testing, primary lesion characteristics, metastatic site specification, imaging examinations, immune therapy commencement timing, PD-L1 expression level, therapeutic regimens, objective response rate (ORR), and disease control rate (DCR). Furthermore, the administration of ICIs prior to treatment and within the interval of 2 to 7 days post-treatment was recorded. Moreover, an extensive panel of peripheral blood parameters was assessed, comprising T lymphocyte subsets, which encompassed CD3^+^%, CD4^+^%, CD8^+^%, CD4^+^/CD8^+^ ratio, CD3^+^ T cell absolute count, CD4 T cell absolute count, CD8^+^ T cell absolute count, Treg/CD4^+^T ratio. The evaluation also extended to myeloid suppressor cell subsets, including M-MDSC/MDSC ratio and G-MDSC/MDSC ratio. The categorization of smoking status was ascertained, with non-smokers defined as individuals who have either smoked fewer than 100 cigarettes in their lifetime or abstained from smoking for a period exceeding 15 years. Efficacy evaluation, in accordance with RECIST version 1.1 and iRECIST, mandated a bi-monthly follow-up for enrolled patients. During these assessments, the status of target lesions was primarily appraised through modalities such as CT and MRI.

The outcomes of the therapeutic interventions were categorized into distinct clinical responses, specifically encompassing disease progression (PD), stable disease (SD), partial response (PR), and complete response (CR). Based on the assessment outcomes, the patient cohort was stratified into two subgroups: the responder group (designated as “R”), comprising individuals demonstrating CR, PR, or SD for a minimum duration of six months, and the non-responder group (designated as “NR”), comprising those who exhibited CR, PR, or SD for less than six months, or alternatively, individuals presenting with PD or fatality during the course of the study.

### 2.3 TCM syndromes

To comprehensively assess alterations in TCM syndromes, the changes occurring one week before and one week after the treatment were observed. The diagnostic criteria and therapeutic modalities for TCM syndromes adhere to the guidelines outlined in “Internal Medicine of Traditional Chinese Medicine,” as published by China Traditional Chinese Medicine Press in 2003 (**Table 1**).

**Table 1:** Elements of TCM syndromes.

Syndrome Differentiation	diagnosis
Cough, white sticky phlegm, thick volume, pale complexion, pale tongue, white greasy coating on the tongue, slippery pulse	Syndrome of phlegm-damp obstructing lung
Yellow phlegm, coarse breathing, phlegm with blood or coughing blood, red face, red tongue, yellow greasy coating on the tongue, slippery pulse	Syndrome of phlegm-heat obstructing lung
Cough, scanty phlegm that is white and sticky, chest tightness, abdominal distension, pale tongue, slow and slippery pulse or wiry and slippery pulse	Syndrome of Qi stagnation and phlegm obstruction
Cough with difficulty in clearing, chest pain, worsened pain at night, dark red sputum with blood, lips appearing purplish or dusky, tongue showing purplish discoloration or ecchymosis, thin tongue, pulse feeling string-like and thin or string-like and rough	Syndrome of phlegm and blood stasis
Fatigue and weakness, reduced speech, loose stools, facial edema and limb swelling, poor sleep at night, pale and tender tongue, deep or weak pulse	Syndrome of spleen-lung Qi deficiency

### 2.4 Sample collection

Stool and serum samples were collected in the morning following an overnight fast of at least 8 hours. Fecal samples were partitioned into five equal portions, each containing 200mg of material, and subsequently deposited into sterile cryogenic tubes, which were then placed in insulated containers with ice packs. These samples were promptly transported to the laboratory and preserved at -80°C. Blood samples were collected to obtain serum. Following blood coagulation, the sample-containing tube was gently agitated, after which centrifugation was conducted at 3000 revolutions per minute (rpm) for a duration of 10 minutes. The resultant supernatant, representing the serum, was then transferred into 1.5ml cryogenic tubes and subsequently preserved at -80°C to facilitate further analytical procedures.

### 2.5 Extraction and PCR amplification of genomic DNA

The extracted DNA was subjected to analysis for integrity and fragment size through 1% agarose gel electrophoresis. Polymerase chain reaction (PCR) amplification was performed using the primers 515F (5 ‘-GTGYCAGCMGCCGCGGTAAa-3’) and 806R (5 ‘-GGACTACNVGGGTWTCTAA-3’). It is worth noting that the 5’ termini of these primers contain specific barcodes corresponding to each individual sample, in addition to a universal sequencing primer. This amplification procedure targeted specific regions of the 16S rRNA gene. The amplified PCR products were then subjected to further analysis via electrophoresis employing a 2% agarose gel. Following verification, magnetic beads were utilized for purification. To initiate the sequencing library construction, the TruSeq^®^ DNA PCR-Free sample preparation kit was utilized. Subsequently, the library was quantified using Qubit and quantitative PCR (Q-PCR). Upon qualification, sequencing was executed utilizing the Illumina NovaSeq platform.

### 2.6 Bioinformatic analysis

#### 2.6.1 Sequencing data preprocessing

According to the barcode sequences and PCR amplification primer sequences, sample-specific information was derived from the demultiplexed data. Subsequently, the barcode and primer sequences were removed, and the sample reads were concatenated using the FLASH (V1.2.7) software to generate Raw Tags data. Following this, the Raw Tags dataset underwent filtration via the fastp software to obtain a subset of high-quality Clean Tags. The Tags sequences were compared against a species annotation database to eliminate chimera sequences (Caporaso et al., 2010), culminating in the acquisition of the Effective Tags dataset.

#### 2.6.2 OTU clustering and species annotation

The Uparse algorithm (Uparse v7.0.1001) was employed to perform Operational Taxonomic Units (OTUs) clustering of the Effective Tags sequence, using a 97% identity threshold as the standard. Subsequently, representative OTUs sequences were selected. These representative sequences underwent annotation by matching them to species in the SSUrRNA database. Taxonomic information was acquired, enabling classification at various taxonomic levels, including kingdom, phylum, class, order, family, genus, and species, facilitating the determination of community composition within the sample. For the establishment of phylogenetic relationships among the OTUs representing sequences from the sample, multiple sequence alignments were executed using the MUSCLE software (Version 3.8.31). Finally, the data was normalized according to the OTUs with the lowest data abundance within the sample.

#### 2.6.3 Sample complexity analysis

The Alpha diversity index was calculated using QIIME software (Version 1.9.1) to analyze the distinctions in Alpha diversity indices across the various groups. To examine the differences in β-diversity indices among groups, R software was employed. LEfSe software was utilized to execute Linear Discriminant Analysis Effect Size (LEfSe) analysis, applying an LDA Score screening threshold of 4. Metastats analysis was conducted using R software. For the inter-group displacement test, data were stratified according to taxonomic ranks, spanning phylum, class, order, family, genus, and species levels. The analysis yielded initial P-values, subsequently subjected to appropriate modifications.

#### 2.6.4 Functional annotation

The complete genome sequences of prokaryotic organisms, encompassing the entire 16S rRNA gene, were retrieved from the KEGG database. Subsequently, these sequences were subjected to comparison with the SILVA database, employing a BLAST bitscore threshold exceeding 1500, thereby establishing a correlation matrix. The functional information associated with the entire prokaryotic genome, as annotated within the KEGG database, was aligned with the corresponding content within the SILVA database, culminating in comprehensive functional annotation of the SILVA database. Conclusively, leveraging the sequences contained within the SILVA database as a reference, the process of OTU clustering for the samples was executed, enabling the accomplishment of functional predictions through Tax4Fun.

### 2.7 Peripheral blood flow cytometry

**Figure 2:**
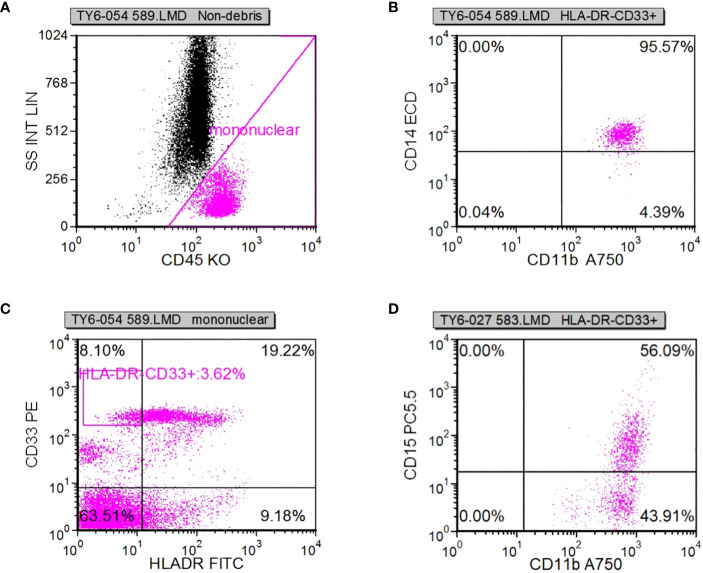
**(A-D)** The idea of detecting myeloid inhibitory cells.

Following specimen qualification and preparation, distinct antibody reagents were individually introduced into the base of the test tube. The specific antibodies employed included HLA-DR-FITC, CD14-ECD, CD15-PC5, CD11b-APC-Alexa Flour 750, and CD45-Krome Orange. For the assessment of T lymphocyte subsets, a volume of 100µL derived from uniformly mixed anticoagulated whole blood at a concentration of 1×10^6^/L was introduced. This mixture was subsequently agitated and incubated in the absence of light for a duration of 15-20 minutes. The red blood cells were dissolved through the addition of OptiLyseC, followed by the introduction of Flow-Count for computerized detection. In the case of Treg (CD25+FOXP3) (%) and myeloid suppressor cell subsets, antibodies were introduced and incubated under conditions devoid of light for 15 minutes. FASC hemolysin was incubated in a light-free environment for 5 minutes. Upon a 5-minute centrifugation, the supernatant was extracted, and PBS Beckerman (PK-31) buffer, supplemented with 0.1% calf serum, was subjected to a 5-minute centrifugation. Following removal of the supernatant, a 1% paraformaldehyde fixative solution was administered, and the resulting samples were evaluated through flow cytometry. Subsequent data analysis was performed using the batch processing function of FCS EXPRESS3.0 software, with the positioning of gates adjusted according to the target cell populations to be assessed. In cell analysis, myeloid suppressor cells were exemplified. Initially, the mononuclear cell population was delineated, followed by the presentation of the mononuclear cell population in the CD45/ss diagram using HLADR/CD33 double parameters, and subsequently circumscribing HLADR-CD33+. Next, CD11b/CD14 dual parameters were employed to exhibit the HLADR/CD33 cell population within the HLADR-CD33+ gate. Finally, CD11b/CD15 dual parameters were applied to showcase the HLADR/CD33 cell population within the HLADR-CD33+ gate (**Figures 2A–D**).

### 2.8 Statistical analysis

Data analysis was conducted using SPSS 25.0 statistical software. Continuous variables following a normal distribution were presented as mean ± standard deviation (x ± s). Group comparisons were carried out utilizing one-way analysis of variance, and *post hoc* pairwise comparisons were conducted using the least significant difference (LSD) -t test. A significance threshold of *p* < 0.05 signified statistical significance.

## 3 Results

### 31 Discussion on the correlation between intestinal microbiome and immunotherapy of NSCLC

#### 3.1.1 Clinical features of patients

This study encompassed a cohort of 39 patients, with an average age of 62 ± 8.15 years. The gender distribution consisted of 30 males and 9 females. Pathological classification revealed 15 cases of non-squamous cell carcinoma and 24 cases of squamous cell carcinoma. Disease staging delineated 17 cases at stage III and 22 cases at stage IV. Metastatic sites were identified, including 13 cases of contralateral lung metastases, 3 cases of liver metastases, 8 cases of brain metastases, 4 cases of renal or adrenal metastases, and 12 cases of bone metastases.

According to iRECTST 1.1 criteria, the distribution of response outcomes in the patient cohort was as follows: 1 case of CR, 13 cases of PR, 10 cases of SD, 12 cases of PD, and 3 recorded deaths. Consequently, the ORR was calculated as 35.9% (14/39), and the DCR was determined to be 61.5% (24/39). There were 24 patients categorized into the responder group (R), with a median age of 63.5 ± 8.65, consisting of 19 males and 5 females. In the non-responder group (NR), there were 15 individuals, comprising 11 males and 4 females, with a median age of 59 ± 7.47 years. There were no statistically significant differences in gender, age, smoking status, disease staging, pathological type, primary lesion, ECOG score, or PD-L1 expression between the two groups (*p* > 0.05). Likewise, other indicators such as surgical history, radiotherapy, and hyperthermia demonstrated no significant differences between the two groups (*p >* 0.05).

In the study, 39 patients underwent pre-treatment sample collection, while 30 patients participated in post-ICIs sample procurement. However, for nine patients, the acquisition or assessment of samples following treatment was infeasible due to external factors. This study was subsequently segregated into ICIs pre-treatment (BT) and post-treatment (AT) groups, each consisting of 30 individuals (n=30 vs. 30). Additionally, the analysis encompassed the segregation of patients into the R group and NR group, comprising 24 and 15 individuals, respectively (n=24 vs. 15). Detailed information is presented in **Table 2**.

**Table 2:** Fundamental patient characteristics.

Patient Characteristics	All (n=39)	R (n=24)	NR (n=15)	*p-*value
Average age (years)				
< 60	20	11 (43.5)	9 (60.0)	0.508
≥ 60	19	13 (56.5)	6 (40.0)	
Gender				
Male	30	19 (79.2)	11 (73.3)	0.711
Female	9	5 (20.8)	4 (26.7)	
ECOG rating				
0	13	5 (20.8)	8 (53.3)	0.280
1	20	13 (54.2)	7 (46.7)	
2	6	6 (25.0)	0	
Smoking history				
Present	23	14 (58.3)	9 (60.0)	> 0.999
Absent	16	10 (41.7)	6 (40.0)	
Histopathological classification				
Squamous cell carcinoma	25	14 (58.3)	11 (73.3)	0.496
Non-squamous cell carcinoma	14	10 (41.7)	4 (26.7)	
Clinical staging				
III	17	12 (50.0)	5 (33.3)	0.343
IV	22	12 (50.0)	10 (66.7)	
Primary lesion				
Right lung	15	10 (66.7)	5 (33.3)	> 0.999
Left Lung	24	14 (62.5)	10 (37.5)	
Location				
Central	15	7 (29.2)	8 (53.3)	0.182
Peripheral	24	17 (70.8)	7 (46.7)	
PD-L1 expression				
Negative	23	14 (58.3)	9 (60.0)	> 0.999
Positive	16	10 (41.7)	6 (40.0)	

#### 3.1.2 Changes of TCM syndromes

The **Supplementary Table 1** in **Supplementary Materials** displays the TCM syndrome types of the included patients before and after treatment. Prior to treatment, spleen-lung Qi deficiency and Qi-Yin deficiency manifested in 30 patients, constituting 76.92% of the cohort. Following treatment, this number decreased to 23 patients, representing 58.97% of the total. Notably, nine patients experienced an improvement in their pathological symptoms post-treatment. Among the patients assessed before treatment, nine patients (23.08%) exhibited syndromes of phlegm-damp obstructing lung, phlegm-heat obstructing lung, Qi stagnation and phlegm obstruction, and phlegm and blood stasis. After treatment, these conditions remained in four patients, while five patients demonstrated amelioration of their pathological symptoms. In patients with advanced NSCLC, the predominant clinical features included spleen-lung Qi deficiency syndrome and Qi-Yin deficiency syndrome. Prior to treatment, spleen-lung Qi deficiency syndrome prevailed as the primary condition in both the R group and NR group, constituting 50.0% and 46.7%, respectively. Qi-Yin deficiency syndrome followed closely, accounting for 29.2% in the R group and 26.7% in the NR group (**Supplementary Table 2** in **supplementary materials**). Notably, the proportion of patients exhibiting these syndromes significantly decreased after treatment. The T-test was employed to assess changes in Traditional Chinese Medicine (TCM) syndromes between the R group and NR group. It was observed that the proportion of patients exhibiting lung-qi deficiency syndrome returning to normal after treatment was greater than those with Qi-Yin deficiency syndrome, although the overall difference was not statistically significant (P>0.05). Subsequently, a t-test was applied to compare the proportion of lung-qi deficiency and Qi-yin deficiency in the R group before and after treatment, revealing no statistical significance (P>0.05). In the NR group, the proportion of lung-qi deficiency and Qi-Yin deficiency decreased before and after treatment, with no statistically significant difference (P>0.05).

#### 3.1.3 OTU analysis

**Figure 3:**
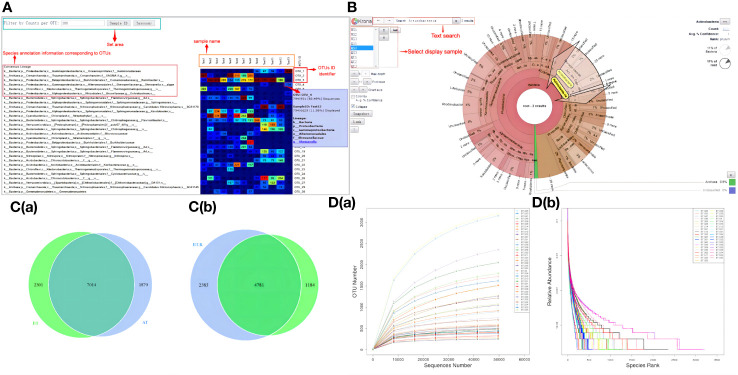
OTU analysis results. **(A)** Heatmap of OTU sequence annotation results. **(B)** Visual representation of species annotation results. **(C)** Venn maps (a) OTU-based Venn map (b) Genes within BT-AT group and R-NR group-based Venn map. **(D)** Curves (a) Dilution curve (b) Rank clustering curve.

Each sample yielded an average of 85,040 original data points, with a subsequent 84.10% effective data rate, resulting in a total of 71,527 high-quality data points following rigorous quality control measures. According to the 97% identity threshold for OTU clustering, a total of 8,403 OTUs were successfully identified. The outcomes of species annotation revealed that all 8,403 OTUs were annotated, with 8,371 OTUs (99.62%) being matched to the database. The proportions of OTU annotations at the taxonomic ranks of kingdom, phylum, class, order, family, genus, and species were 99.62%, 82.71%, 79.66%, 70.82%, 57.72%, 34.36%, and 6.55%, respectively. **Figure 3A** illustrates the interactive web page depicting the species annotations of OTUs, offering a visual representation of the species composition and their respective abundance within the sequenced samples. Meanwhile, **Figure 3B** employs KRONA visualization to present the species annotation results graphically.

The OTU results revealed that the total gene count for the BT group and the AT group amounted to 7,014 genes. Specifically, the BT group possessed 2,301 unique genes, while the AT group featured 1,879 unique genes. Notably, despite both groups consisting of samples obtained from the same cohort of patients, a substantial variance in the gene composition of the samples was observed, indicating the influence of ICIs treatment on the gut microbial gene profiles in patients with NSCLC (**Figure 3C**). Similarly, the total gene count for the responder group before treatment (BT.R) and the non-responder group before treatment (BT.NR) was 4,781 genes. Among these, 2,385 genes were distinct to the BT.R group, whereas 1,184 genes were exclusive to the BT.NR group. Given the uniformity of advanced NSCLC within the samples, the intestinal microbiomes of these cancer subtype exhibited a high degree of genetic similarity, suggesting the presence of shared tumor-related genetic markers (**Figure 3C**).

In general, the experimental samples exhibited commendable and rational quality, characterized by a noteworthy degree of species abundance and uniformity, as visually depicted in **Figure 3D**.

#### 3.1.4 Relative abundance of species

Based on the species annotations, species with higher relative abundance within each group were sorted across various taxonomic levels, including phylum, class, order, family, genus, and species. Subsequently, relative abundance histograms were constructed, highlighting the top three species with the highest proportions.

##### 3.1.4.1 Relative abundances of species in the BT and AT groups

**Figure 4:**
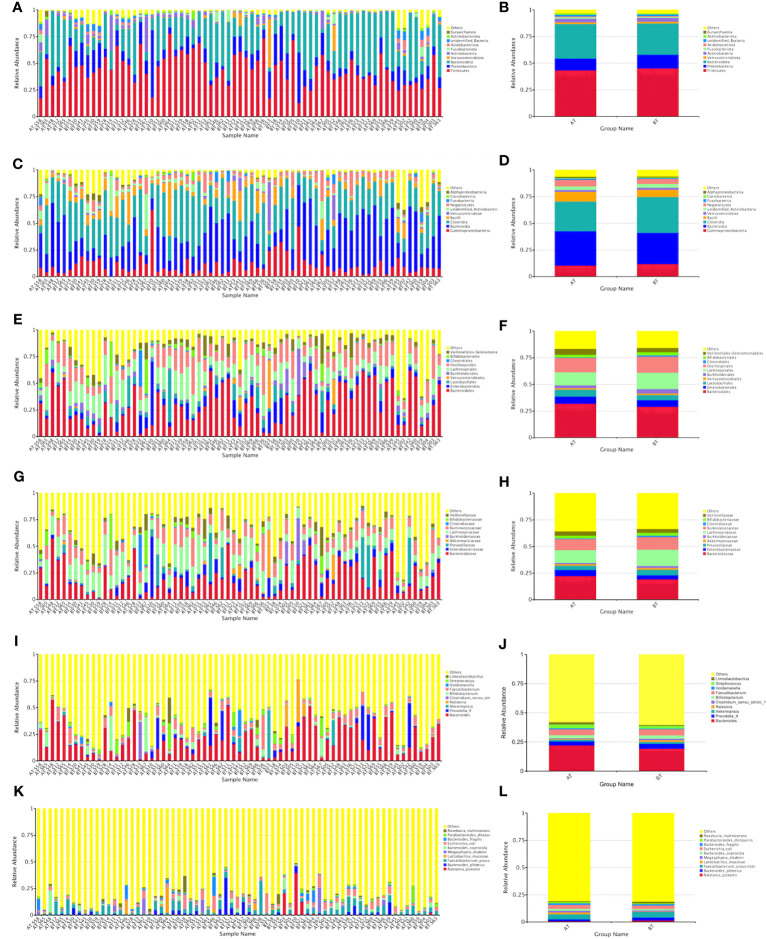
Bar chart of relative abundance of species at various taxonomic levels (phyla **A, B**, class **C, D**, order **E, F**, family **G, H**, genus **I, J**, and species **K, L**).

At the levels of phylum, class, order, family, genus, and species, the top three in terms of relative abundance were as follows: Firmicutes, Bacteroidetes, and Proteobacteria (**Figures 4A, B**); Proteobacteria, Bacteroidetes, and Clostridium (**Figures 4C, D**); Bacteroidei, Enterobacteriformes, and Lactobacilli (**Figures 4E, F**); Bacteroideaceae, Enterobacteriaceae, and Prevotellaceae (**Figures 4G, H**); Bacteroides, Prevotella, and Akkermansia mucinophilus (**Figures 4I, J**); Rolstoniella pictori, Bacteroides vulgaris, Clostridium tenuensis (**Figures 4K, L**).

##### 3.1.4.2 Relative abundances of species in the R and NR groups after ICIs treatment

**Figure 5:**
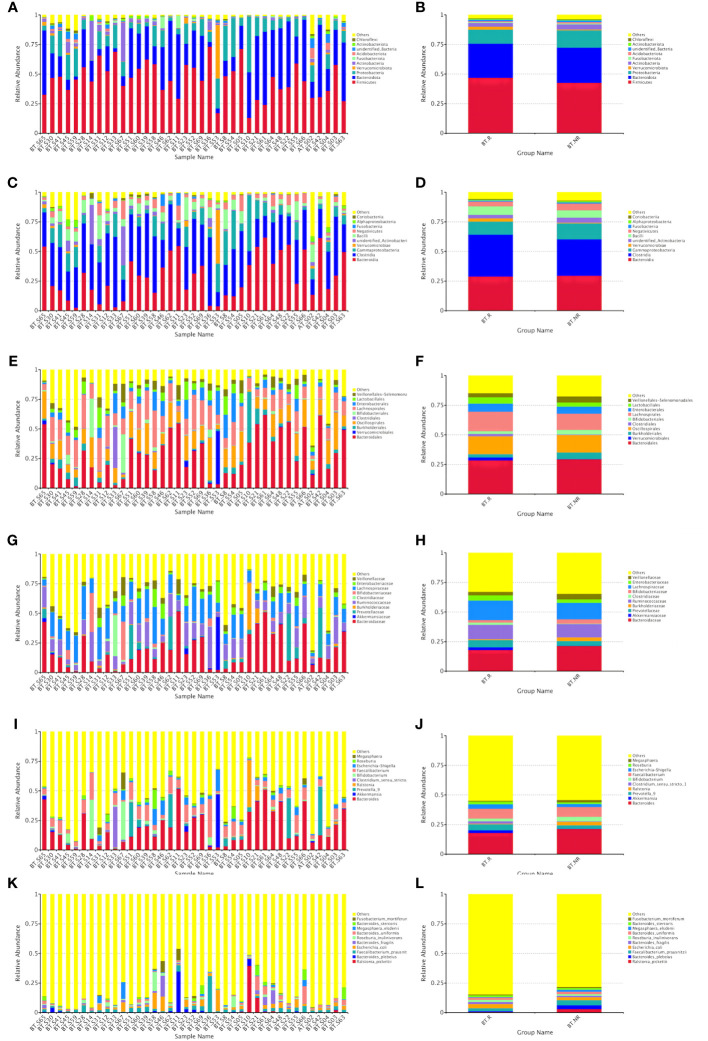
Bar chart of relative abundance of species treated by ICIs at various taxonomic levels (phyla **A, B**, class **C, D**, order **E, F**, family **G, H**, genus **I, J**, and species **K, L**).

At the phylum, class, order, family, genus, and species levels, the top three in terms of relative abundance were as follows: Firmicutes, Bacteroidetes, and Proteobacteria (**Figures 5A, B**); Bacteroideta, Clostridium, and Proteobacteria (**Figures 5C, D**); Bacteroideae, Verrucomicrobacteriales, and Burkella (**Figures 5E, F**); Bacteroideaceae, Ackermanniaceae, and Prevotellaceae (**Figures 5G, H**); Bacteroides, Akkermansia mucophil, and Prevotellaceae (**Figures 5I, J**); Rolstoniella picti, Bacteroides vulgaris, and Clostridium tenuis (**Figures 5K, L**).

#### 3.1.5 Analysis of differences between groups

##### 3.1.5.1 Inter-group difference analysis of Alpha diversity index

We investigated the distribution of the total number of species in various sample groups and conducted inter-group analysis to compare differences in sample diversity and uniformity.

Following ICIs treatment, the α diversity indices, including the observed-species index and Chao-1 index, exhibited a general increase. However, statistical analysis using both the T test and Wilcoxon rank-sum test revealed that the Shannon, Simpson, Chao-1 indices of patients in both groups demonstrated significant increases after treatment. Moreover, the Chao-1 P values exceeded 0.05, signifying a lack of statistical significance between the groups. These results indicate that there were no significant differences in colony richness and species diversity between the AT and BT groups (**Table 3**).

**Table 3:** Comparison of immune parameters in patients with different therapeutic effects after treatment (X ± S).

Index	Post-treatment		T-value	*p*-value
	NR (n=10)	R (n=20)		
CD3^+^(%)	69.01 +/- 7.05	77.03 +/- 10.98	2.093	0.046
CD4^+^(%)	35.70 +/- 4.59	41.15 +/- 6.40	2.391	0.024
CD8^+^(%)	30.90 +/- 6.35	25.75 +/- 5.83	2.218	0.035
CD4^+^/CD8^+^(%)	1.37 +/- 0.37	1.69 +/- 0.38	2.179	0.038
CD3^+^	533.12 +/- 236.95	665.86 +/- 402.17	0.959	0.346
CD4^+^	293.74 +/- 153.62	335.28 +/- 195.17	0.587	0.562
CD8^+^	226.07 +/- 127.84	204.73 +/- 129.76	0.427	0.673
Treg/CD4^+^	4.99 +/- 0.80	4.40 +/- 0.70	2.119	0.043
M-MDSC/MDSC	4.42 +/- 9.13	1.84 +/- 2.31	1.208	0.237
G-MDSC/MDSC	0.65 +/- 1.30	0.43 +/- 0.80	0.573	0.571

Similarly, following ICIs treatment, the α diversity indices, including the Shannon, Simpson, and Chao-1 indices, displayed a slight elevation in group R compared to group NR. However, the results of the T and Wilcoxon rank-sum tests revealed that the Shannon, Simpson, and Chao-1 P values all exceeded 0.05, indicating a lack of statistical significance. This suggests that there were no notable distinctions in colony richness and species diversity between the BT.R and BT.NR groups.

##### 3.1.5.2 Inter-group difference analysis of β-diversity index

**Figure 6:**
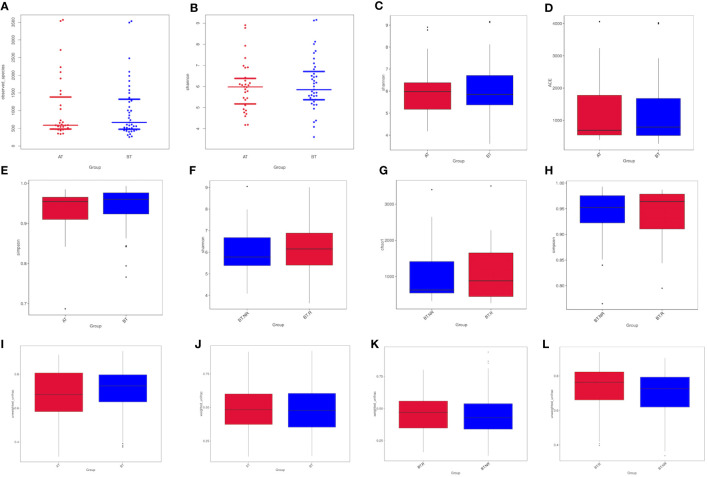
Analysis of Alpha diversity indices between groups. **(A, B)** Differences in observed species between the AT and BT groups. **(C-E)** Differences in the Shannon, Chao-1, and Simpson indices between the AT and BT groups; **(F-H)** Differences in the Shannon, Chao-1, and Simpson indices between the R and NR groups. **(I, J)** Differences in the AT and BT groups based on weighted/unweighted UniFrac β-diversity. **(K, L)** Differences in the R and NR groups based on weighted/unweighted UniFrac β-diversity.

Upon comparing the samples before and after receiving ICIs treatment, a noticeable reduction in the β-diversity index was observed post-treatment, signifying significant differences in microbial communities between the AT and BT groups. The weighted UniFrac-T test and Wilcoxon rank-sum test yielded P-values of 0.970 and 0.743, respectively. These statistical analyses indicated the absence of significant differences in β-diversity between the pre-treatment and post-treatment groups, implying that the constituent species remained consistent (**Figures 6A–H**).

When comparing the R and NR groups, it was observed that the β-diversity index of the R group increased, signifying a significant difference in microbial community composition between these groups with respect to ICIs treatment. The weighted Unifrac-T test and rank-sum test yielded P-values of 0.213 and 0.146, respectively. The statistical analyses indicated the absence of significant differences in β-diversity between the R and NR groups (**Figures 6I–L**).

##### 3.1.5.3 Weft drop analysis

**Figure 7:**
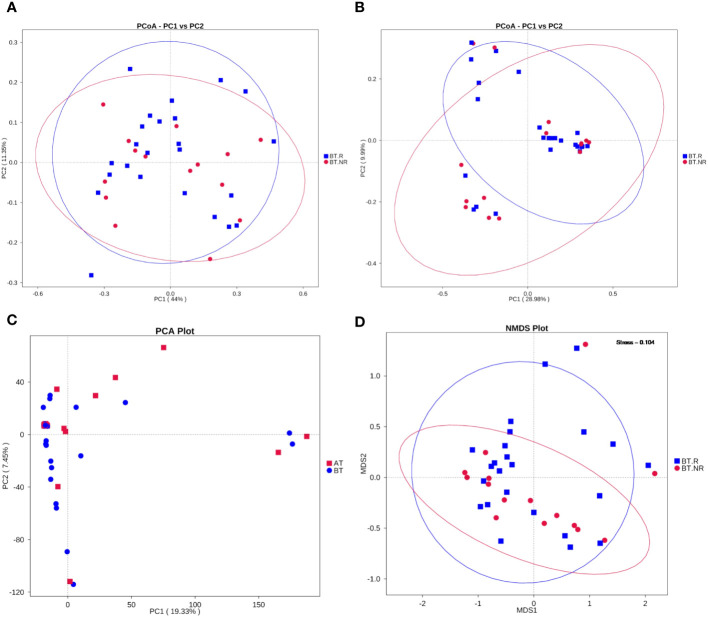
Results of dimensional reduction analyses. **(A, B)** PCoA analysis based on weighted/unweighted Unifrac distances between R and NR groups; **(C)** PCA analysis of inter-group R-NR variations. **(D)** NMDS analysis.

To further explore the similarity in species composition among groups, we employed principal coordinates analysis (PCoA), principal component analysis (PCA), and non-metric multi-dimensional scaling (NMDS). The results of PCoA revealed that the species structure of the ICIs-treated R group closely resembled that of the NR group. Most samples within the BT.R and BT.NR groups displayed substantial homogeneity, whereas the BT.R group exhibited notable structural variation, with the BT.NR group displaying minimal divergence. Principal components PC1 and PC2 contributed 28.98% and 9.99%, respectively, to the dissmilarity among samples (**Figures 7A, B**). NMDS analysis corroborated the findings of PCoA (**Figure 7D**). Furthermore, PCA analysis demonstrated that the samples within the BT.R and BT.NR groups were positioned in closer proximity to one another, while individual distances were extended. This suggested a degree of similarity in community composition between and within the two groups. The first principal component PC1 and the second principal component PC2 contributed 24.36% and 10.15%, respectively, to the distinctions among samples (**Figure 7C**).

#### 3.1.6 Analysis of species significance difference

Through T-test analysis and LEfSe analysis, we conducted an exploration of species exhibiting significant variations in abundance between the R_NR and BT_AT groups. This allowed us to identify the enrichment of distinct species in each group. Simultaneously, we compared differences between the R_NR and BT_AT groups to assess the significance of disparities in community structure.

To pinpoint species with notable differences between groups (p<0.05), we initiated the analysis from the perspective of species abundance at various taxonomic levels (Phylum, Class, Order, Family, Genus, Species). Biomarkers were identified through inter-group T-tests.

##### 3.1.6.1 Species difference analysis between BT group and AT group(T-test)

In comparison with the AT group, the relative species abundance within the BT group did not exhibit significant changes at the phylum, class, and order levels. However, at the family level, there were 9 species with significant differences in the abundance between the AT and BT groups. Notably, six species of Pseudoalteromonadaceae, Alcaligenaceae and Neisseriaceae all displayed decreased abundance following ICIs treatment. Three additional families, Micropepsaceae, Parvibaculaceae and Methylococcaceae, disappeared after ICIs treatment. At the geneus level, 20 strains within the AT and BT groups showed significant changes in abundance. Of these, 11 strains, such as Roseburia, Pseudoalteromonas, and Dorea, experienced a decrease in relative abundance after treatment, while four strains, including Dielma, exhibited an increase in relative abundance after ICIs treatment. Moreover, five strains, including Psychrobacter and Marinospirillum, disappeared after ICIs treatment. At the species level, 11 species within the AT and BT groups displayed significant changes in abundance. Specifically, nine species in the AT and BT groups, including Roseburia_inulinivorans, Parabacteroides_merdae, and Dorea_formicigenerans, all experienced a decrease in abundance following ICIs treatment. On the other hand, the species Acinetobacter increased in abundance after ICIs treatment, whereas Psychrobacter_alimentarius and Marinospirillum_minutulum disappeared following ICIs treatment.

Data pertaining to bacteria exhibiting significant differences before and after ICIs treatment is presented in **Supplementary Table 3** in **Supplementary Materials**. This includes the mean abundance (> 0.01%) of each species, as well as the corresponding significance *P-values*, which were calculated using the T-test to assess the variation between the two groups.

##### 3.1.6.2 Analysis of species differences between R and NR groups(T-test)

At the phylum level, no significant differences were observed in the species abundance between the R and NR groups treated with ICIs. However, at the order level, noteworthy variations were evident, with five species exhibiting significant differences between the R and NR groups. Notably, Monoglobales and Cardiobacteriales experienced an enrichment in the R group, with Cardiobacteriales displaying the highest average abundance (2.52). Conversely, Acetobacterales exhibited increased enrichment in the NR group, boasting an average abundance of 5.37. Moving to the family level, 14 strains displayed significant differences in abundance between the R and NR groups. Among these, 11 bacteria families, such as Butyricicoccaceae, Cardiobacteriaceae (2.52), and the unidentified Clostridia UCG-014 (6.72), experienced substantial enrichment in the R group. Cardiobacteriaceae (2.52), Parachlamydiaceae, and the unidentified Clostridia UCG-014 (6.72) are exclusively enriched in the R group, while Catenulisporaceae (6.72), Spirosomaceae (4.03), and Acetobacteraceae (5.37) were most abundant in the NR group. At the genus level, 24 bacteria species exhibited significant differences in abundance between the R and NR groups. Among these, 20 genera, such as Sinomonas and Cardiobacterium (2.52), displayed increased enrichment in the R group. Conversely, four species, including unidentified Clostridia UCG-014 (6.72) and Actinocatenispora (5.88), were uniquely enriched in the R group. Terracidiphilus (6.72), Catenulispora, Lactococcus, and GCA-900066755 (5.37) were notably more abundant in the NR group. Finally, at the species level, 13 strains within the R and NR groups exhibited significant differences in abundance, with 12 of these species, such as Terrabacter_sp and Sinomonas_atrocyanea, showcasing greater enrichment in the R group. Notably, Clostridiales_bacterium_oral_taxon_075 (6.72), Faecalitalea_cylindroides, and Pseudarcicella_hirudinis were exclusively observed in the R group. Conversely, Blautia_hydrogenotrophica (6.72) exhibited higher abundance in the NR group.

##### 3.1.6.3 LEfSe

**Figure 8:**
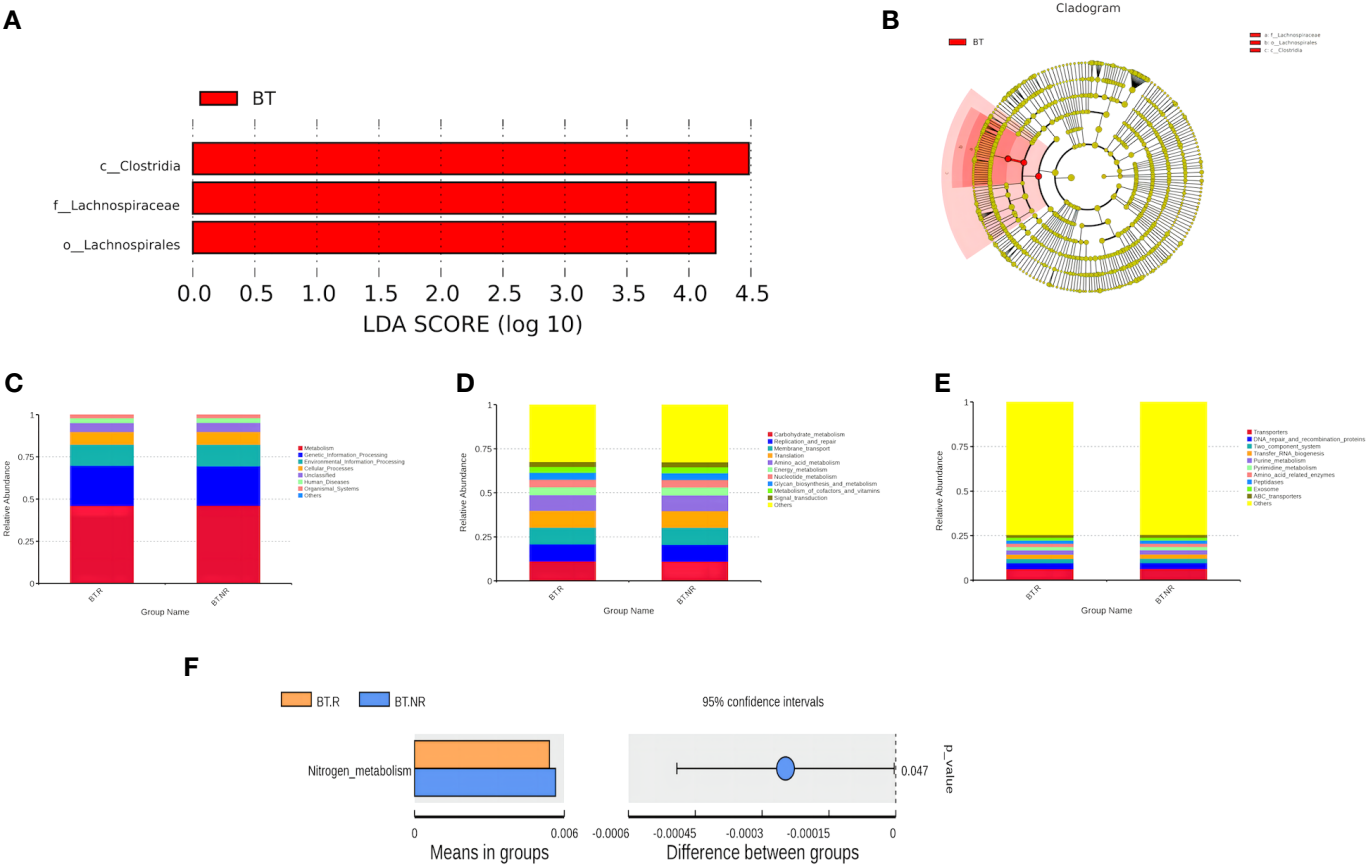
**(A, B)** Distribution bar chart of LDA values and evolutionary branching chart. **(C-F)** Bar plots depicting relative abundance of functional annotations at Tax4Fun functional note levels 2 and 3.

Employing LEfSe analysis (Segata et al., 2011), we conducted a comprehensive exploration of biomarkers exhibiting significant differences between the AT and BT groups. The results have unveiled three distinct bacterial strains with notable variations in their abundance profiles, qualifying them as prospective biomarkers. These strains are specifically recognized as Clostridia, Lachnospiraceae, and Lachnospirales. Within the BT group, we identified three distinct biomarkers characterized by LDA values surpassing 4, which were precisely denominated as Clostridiae, Trichospirillaceae, and Trichospirales. Intriguingly, an intricate hierarchical relationship was observed among these biomarkers, with the order of precedence being Lachnospiraceae<Lachnospirales<Clostridia. Notably, Clostridia belonged to the clade of Firmicutes (**Figures 8A, B**).

#### 3.1.7 Function prediction

**Figure 9:**
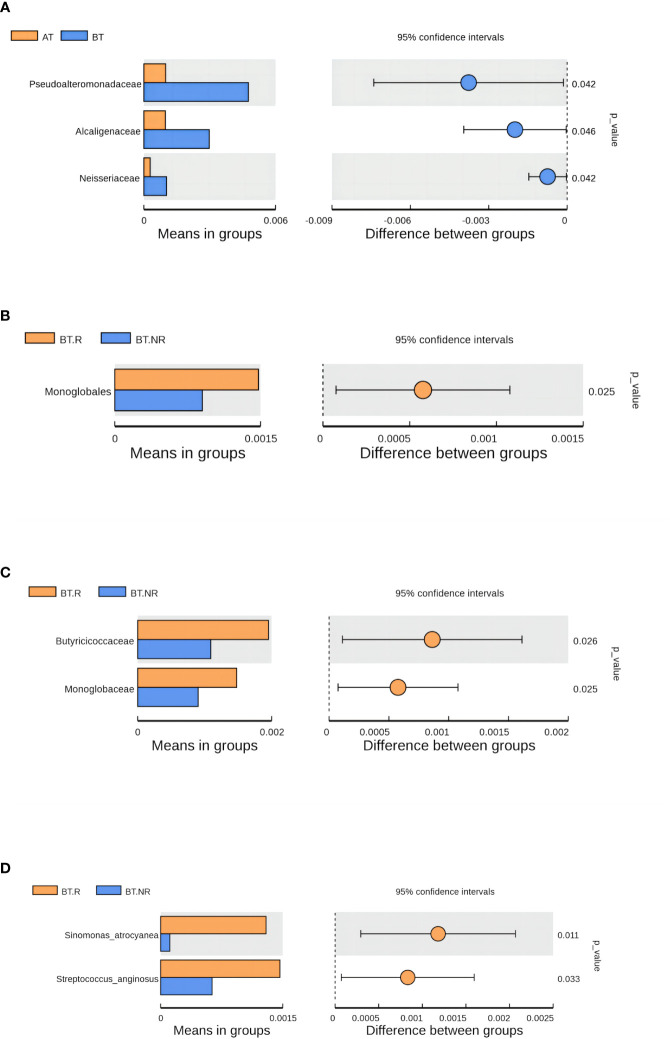
Analysis results of species differences before and after treatment. **(A)** Species differences between AT and BT groups; **(B-D)** Species differences between R and NR groups.

Tax4Fun was employed to predict the function of Operational Taxonomic Units (OTUs) within the BT.R_BT.NR groups. Based on the database interpretation results and the T-test difference analysis, a bar chart illustrating the top 10 functional information at each classification level within the group was generated (**Figures 9A, B**).

Approximately 50% of the functional attributes within the BT.R and BT.NR groups undergoing ICIs treatment were associated with metabolic processes, including amino acid metabolism, nucleotide metabolism, among others. In addition, functions related to environmental information processing, genetic information processing, and cellular processes were prominent, as well as those linked to cellular transport and transporter functions. Nevertheless, based on the T-test, no statistical significance was observed between the two groups (*p > 0.*05). However, a noteworthy distinction emerged in the relative abundance of nitrogen metabolism between the R and NR groups (*p* < 0*.05*), with a lesser proportion observed in the R group and a more substantial proportion in the NR group (**Figures 8C–F**).

### 3.2 Effects of immunotherapy on the immune microenvironment of NSCLC patients

#### 3.2.1 Effects of ICIs on the immune microenvironment of NSCLC patients

**Figure 10:**
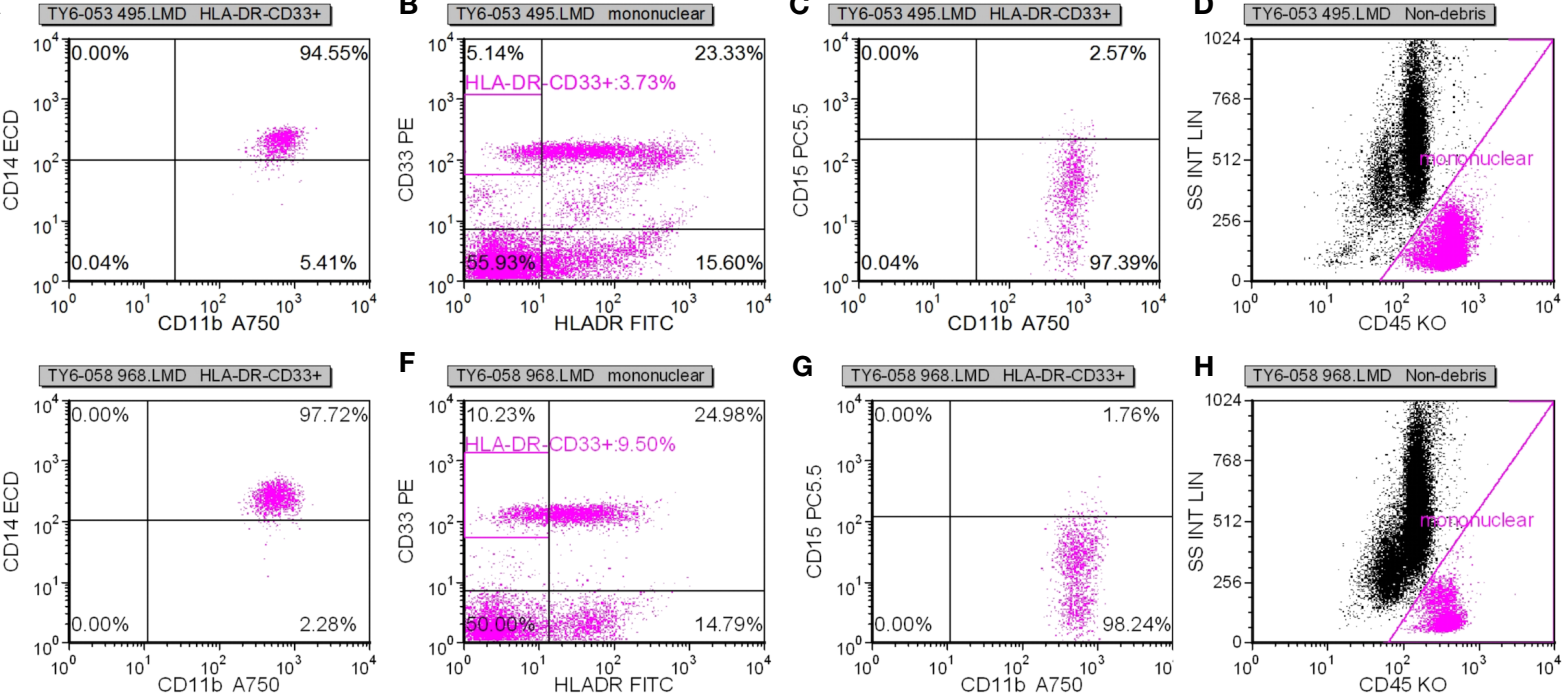
**(A-H)** Changes of myeloid granulocyte subsets before and after ICIs treatment.

**Figure 11:**
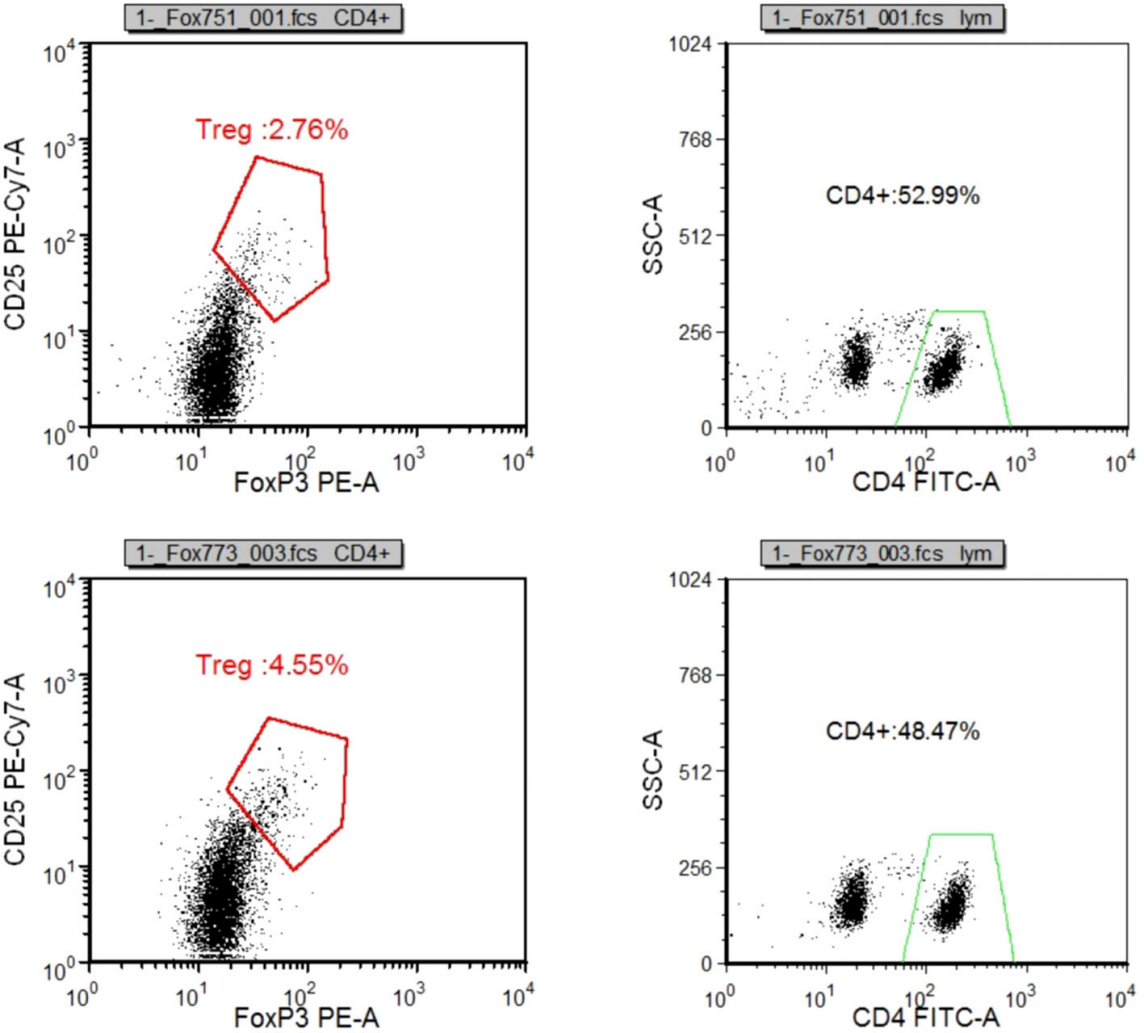
Treg accounted for CD4+ cells before and after ICIs treatment.

Compared to the BT group, the AT group exhibited a significant increase in CD3^+^%, CD4^+^%, and CD4^+^/CD8^+^ ratio within T lymphocyte subsets. In contrast, CD8^+^T% and Treg/CD4^+^ ratio decreased significantly, along with a notable reduction in the absolute count of CD3^+^T cells and CD8^+^T cells (*p* < 0.05). There were no substantial changes in the absolute count of CD4^+^T cells (*p* > 0.05). In the myeloid granulocyte subsets, both M-MDSC/MDSC and G-MDSC/MDSC ratios decreased after ICIs treatment (**Figures 8A**, **10**, **11**, **Supplementary Table 5** in **Supplementary Materials**).

#### 3.2.2 Effect of ICIs on immune microenvironment of NSCLC patients with different therapeutic efficacy

**Figure 12:**
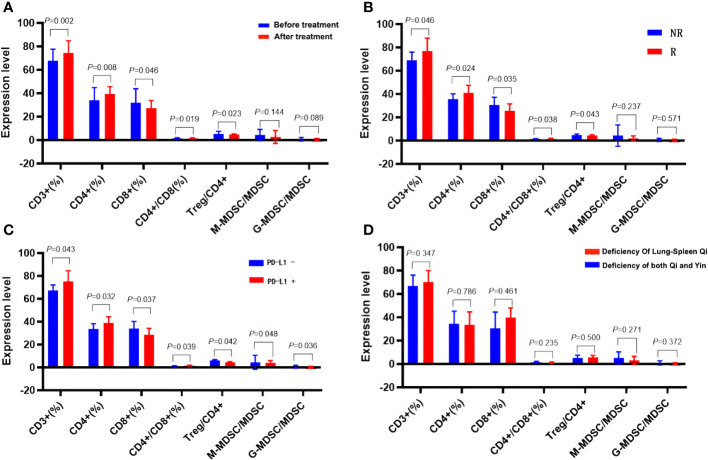
Effects of ICIs on peripheral immune parameters. **(A)** Pre- and post- treatment comparison. **(B)** Comparison between R group and NR group patients. **(C)** Patients with differing PD-L1 expression. **(D)** Patients with different TCM syndromes.

According to the efficacy evaluation, there were 20 patients classified as responders (R) and 10 patients as non-responders (NR), resulting in a treatment effective rate of 67%. There were no statistically significant differences in baseline characteristics such as age, gender, smoking status, ECOG score, and relevant parameters between the R and NR groups (*p > 0.*05). Furthermore, no significant distinctions were observed between the R and NR groups regarding chemotherapy, radiotherapy, hyperthermia, or intervention (*p* > 0.05). Before initiating ICIs treatment, no substantial disparities were found in the ratios of CD3^+^, CD4^+^, CD8^+^ and CD4^+^/CD8^+^, Treg/CD4^+^, M-MDSC/MDSC, and G-MDSC/MDSC between the R and NR groups (*p > 0.*05) (**Supplementary Table 6** in **Supplementary Materials**; **Figure 12B**). However, after ICIs treatment, the R group exhibited significant increases in the CD3^+^%, CD4^+^%, and CD4^+^/CD8^+^ ratio, accompanied by a significant decrease in CD8% and Treg/CD4^+^ ratio, in contrast to the NR group (*p* < 0.05). In the context of immune parameters, the NR group demonstrated higher absolute counts of CD3^+^, CD4^+^, and CD8^+^ T cells compared to the R group before treatment (*p* > 0.05). After treatment, the R group exhibited an increase in absolute counts of CD3^+^ and CD4^+^ T cells, while *the absolute count of* CD3^+^ *and* CD8^+^ T cells significantly decreased compared to the pre-treatment values *(p* < 0.05). Additionally, post-treatment M-MDSC levels significantly decreased in the R group, while G-MDSC levels significantly decreased in the NR group (*p* < 0.05) (**Supplementary Table 6** in **Supplementary Materials**; **Figure 12B**).

#### 3.2.3 Influence of immune microenvironment in NSCLC patients with different PD-L1 expression

Following analysis, eight patients exhibited PD-L1 positivity (PD-L1+, 40%), while 12 patients showed PD-L1 negativity (PD-L1-, 60%). Prior to ICIs treatment, PD-L1+ patients displayed higher serum CD3^+^%, CD4^+^%, and CD4^+^/CD8^+^ ratios, alongside lower CD8%, Treg/CD4^+^, M-MDSC/MDSC, and G-MDSC/MDSC parameters when compared to PD-L1- patients (*p* < 0.05). Furthermore, PD-L1+ patients exhibited higher absolute counts of immune parameters for CD3^+^ and CD4^+^ T cells, but lower counts of CD8^+^ T cells than PD-L1- patients, although these differences did not reach statistical significance (*p* > 0.05) (**Table 4**, **Figures 12A–D**).

**Table 4:** Comparison of peripheral immune parameters in patients with different PD-L1 expression (X ± S).

Index	Expression of PD-L1		T-value	*p*-value
	Low (n=12)	High (n=8)		
CD3^+^(%)	67.12 +/- 5.05	75.03 +/- 9.48	1.693	0.043
CD4^+^(%)	33.70 +/- 4.59	39.15 +/- 5.20	2.031	0.032
CD8^+^(%)	33.90 +/- 6.35	28.75 +/- 5.43	1.518	0.037
CD4^+^/CD8^+^(%)	1.17 +/- 0.37	1.59 +/- 0.38	2.079	0.039
CD3^+^	690.12 +/- 180.95	752.16 +/- 310.13	0.753	0.436
CD4^+^	362.74 +/- 163.62	389.28 +/- 184.12	0.577	0.543
CD8^+^	365.07 +/- 157.74	332.73 +/- 179.76	0.327	0.473
Treg/CD4^+^	6.01 +/- 0.80	4.50 +/- 0.70	2.012	0.042
M-MDSC/MDSC	4.52 +/- 6.12	3.84 +/- 2.21	1.028	0.048
G-MDSC/MDSC	0.65 +/- 1.23	0.38 +/- 0.70	0.533	0.036

#### 3.2.4 Effects of ICIs on the immune microenvironment of NSCLC patients with different TCM syndromes

Patients diagnosed with advanced lung cancer predominantly exhibit Zheng-deficiency. According to the syndrome classification of traditional Chinese medicine, the included patients were categorized into two primary groups: those with spleen-lung Qi deficiency syndrome (19 cases) and those with Qi-Yin deficiency syndrome (11 cases). Among these, the R group consisted of 11 cases with spleen-lung Qi-deficiency syndrome, exhibiting a treatment effective rate of 55%, while the NR group included 9 cases with Qi-Yin deficiency syndrome, achieving a treatment effective rate of 82%. Comparative analysis between the spleen-lung Qi-deficiency syndrome and Qi-Yin deficiency syndrome groups revealed distinct immunological variations. In the latter group, elevated values of CD3^+^% and Treg/CD4^+^ before and after treatment were observed, while CD4%, CD4^+^/CD8^+^, M-MDSC/MDSC, and G-MDSC/MDSC values decreased. These findings suggest a more favorable improvement in the immune microenvironment of patients with Qi-Yin deficiency syndrome following treatment. After treatment, the CD8% value substantially decreased in the Qi-Yin deficiency syndrome group, alongside an increased Treg/CD4^+^ ratio. Notably, values of CD3^+^%, CD4^+^%, and CD4^+^/CD8^+^ in T lymphoid subsets showed an increase before and after treatment, with concomitant reductions in CD8%, Treg, M-MDSC/MDSC, and G-MDSC/MDSC values across both groups (**Supplementary Table 7** in **Supplementary Materials**; **Table 5**, **Figure 9D**).

**Table 5:** Comparison of immune parameters between spleen-lung Qi deficiency syndrome and Qi-Yin deficiency syndrome (X ± S).

Index	Post-treatment		T-value	*p*-value
	Spleen-lung Qi deficiency syndrome (n=19)	Qi-Yin deficiency syndrome (n=11)		
CD3^+^ (%)	74.63 +/- 10.17	73.88 +/- 11.41	0.186	0.854
CD4^+^ (%)	40.29 +/- 7.72	37.67 +/- 2.14	1.093	0.284
CD8^+^ (%)	28.37 +/- 6.61	25.89 +/- 5.97	1.026	0.314
CD4^+^/CD8^+^(%)	1.65 +/- 0.45	1.47 +/- 0.29	1.192	0.243
CD3^+^	643.56 +/- 396.20	583.70 +/- 290.73	0.436	0.666
CD4^+^	333.06 +/- 190.57	301.34 +/- 169.38	0.457	0.651
CD8^+^	290.62 +/- 247.09	250.81 +/- 144.57	0.486	0.631
Treg/CD4^+^	4.54 +/- 0.983	4.70 +/- 0.70	0.555	0.584
M-MDSC/MDSC	1.37 +/- 2.04	5.00 +/- 8.54	1.785	0.085
G-MDSC/MDSC	0.55 +/- 1.17	0.43 +/- 0.53	0.314	0.756

#### 3.2.5 Association of Alpha diversity with immune microenvironment

**Figure 13:**
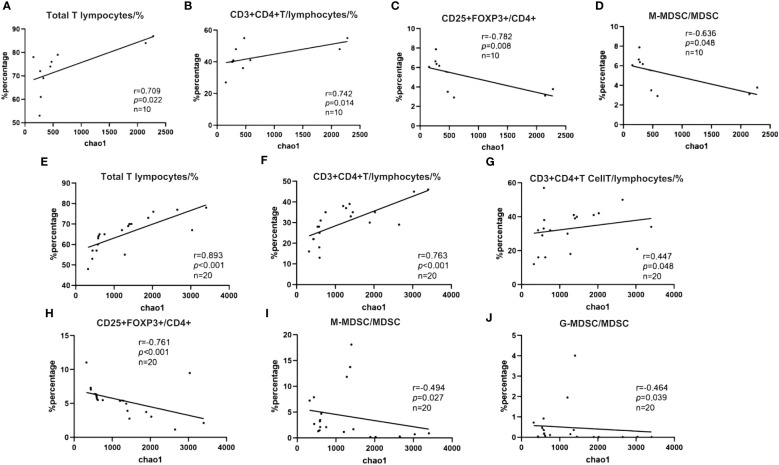
**(A–J)** Spearman correlation between Shannon diversity index and immune characteristics. **(A)** NR group; **(B)** R group.

We analyzed the correlation between the Alpha diversity index of intestinal microorganisms and immune cell parameters within the immune microenvironment. Our findings revealed significant correlations between the Chao-1 index and several immune cell subsets. In the NR group, the Chao-1 index exhibited a positive correlation with CD3% and CD4%, while displaying a negative correlation with Treg/CD4^+^ and M-MDSC/MDSC (*p* < 0.05). Similarly, in the R group, Chao-1 index demonstrated positive correlations with CD3%, CD4%, and CD8%, whereas it exhibited negative correlations with Treg/CD4^+^, M-MDSC/MDSC, and G-MDSC/MDSC (*p* < 0.05). These findings suggest that patients who exhibit a favorable response to ICIs possess not only a well-balanced intestinal flora but also a more favorable immune microenvironment (**Figures 13A–J**).

## 4 Discussion

According to traditional Chinese medicine theory, the lung and the large intestine are intricately linked in both physiological and pathological aspects. Traditional Chinese medicine emphasizes the concept of “lung and large intestine stand in exterior-interior relationship” and “the lung unites with the large intestine and the large intestine is the bowel of conducting through the pathways.” The meridian circulation in the body, specifically the meridian of hand Taiyin lung and the meridian of hand Yangming large intestine, establish mutual collateral connections, facilitating the interplay between the exterior and interior, as well as the flow of pulse and Qi. The lung is classified among the “five zang-organs,” while the large intestine belongs to the “six fu-organs,” and they can exert reciprocal influences on each other in pathological conditions. Modern Chinese medicine has undertaken extensive investigations into the mechanisms of lung and gastrointestinal diseases. Furthermore, a growing body of evidence supports the close association between alterations in the composition of intestinal flora and the onset and progression of cancer (Schwabe and Jobin, 2013; Garrett, 2015). Clinical studies have unveiled disruptions in intestinal flora composition, such as reduced Bifidobacteria levels and imbalanced Bifidobacteria/E. coli ratio, in children with asthmatic bronchitis during the early stages of the disease (Fang et al., 2014). In the context of the ulcerative colitis model, it is observed that dysbiosis within the intestinal microbiota can result in alterations in lung microbiota and the onset of pathological lung damage (Wang et al., 2014). Immunotherapy, a therapeutic approach employed to augment anti-tumor immune responses through the passive or active targeting of tumor cells, aligns with the fundamental principle of “Fuzheng Quxie” in TCM treatment.Extensive research data have illuminated that certain TCM individual remedies, compound formulations, and bioactive constituents can modulate tumor immune responses and exhibit anti-tumor properties through their regulatory actions on the intestinal flora (Li et al., 2020; Lin et al., 2021). Based on the TCM syndrome elements, the patients included in this study were categorized into distinct TCM syndrome types. Within the cohort of advanced NSCLC patients, the predominant syndromes observed were spleen-lung Qi deficiency and Qi-Yin deficiency. The present investigation was undertaken to scrutinize variations in the immune microenvironment among lung cancer patients with different TCM syndrome types. Notably, no significant divergence in immune microenvironment profiles was discerned between the spleen-lung Qi deficiency syndrome and Qi-Yin deficiency syndrome groups. However, it merits mention that the group characterized by Qi-Yin deficiency syndrome exhibited a notably higher treatment response rate compared to the spleen-lung Qi deficiency group. At present, the rationale for this discrepancy remains elusive, primarily because, at the advanced stages of cancer progression, the prevailing syndromes tend to encompass a blend of Qi and Yin deficiencies, phlegm accumulation, and blood stasis. These complex syndrome dynamics do not exhibit a direct correlation with treatment outcomes. We postulate that the variation in treatment efficacy could be linked to the potential dissimilarities in gut microbiota profiles between patients afflicted by syndrome of Yin-deficiency and phlegm-heat, as well as those with syndrome of spleen-deficiency and phlegm-dampness. Nonetheless, this supposition necessitates further rigorous experimental validation to substantiate these conjectures.

We conducted a comprehensive analysis by employing 16S rRNA amplicon sequencing techniques to investigate the intestinal microbiota in patients afflicted with advanced NSCLC. Our primary objective was to elucidate distinctive characteristics associated with the response to ICIs treatment and its associated efficacy. The results derived from the OTUs clustering exhibited a substantial degree of commonality in the gene composition between the BT and AT groups, accounting for over 60%. Furthermore, the genes overlapping between efficacy stratifications represented a noteworthy portion, constituting two-thirds of the total. Of particular note, the R group displayed a higher number of unique OTUs, which provided compelling evidence that ICIs treatment indeed exerts a discernible influence on the patients’ intestinal microbiota. The observations further indicated inter-individual variations in this microbial milieu. It is noteworthy that despite these variations, the gene similarity within the intestinal microbiota remained high, contributing to the overall stability in the structural composition of the gut microbiota. To furnish a comprehensive taxonomy, we conducted a species annotation based on the OTUs sequences by cross-referencing the Silva138 database. This annotation process was carried out across five hierarchical classification levels, including phylum, class, and order. Within this dataset, the dominant bacterial phyla in terms of abundance were Firmicutes, Bacteroidota, and Proteobacteria. Subsequent comparisons between the BT group and the AT group unveiled varied shifts in the relative abundance of distinct bacterial groups. This signifies a notable interaction between ICIs treatment and the intestinal microbiota. Nevertheless, it is important to underscore that the fundamental structural integrity of the gut microbiota largely retained its stability throughout the pre-treatment and post-treatment stages, irrespective of the sustainability of therapeutic benefits.

The profiling of intestinal flora attributes in patients afflicted with varying grades of lung adenocarcinoma, as per the new grading paradigm, assumes paramount importance for the precise formulation of therapeutic strategies and the effective management of prognostic outcomes (Moreira et al., 2020; Deng et al., 2021). In the current landscape of lung cancer, and indeed, across a spectrum of malignancies, certain microbial strains have emerged as exhibiting either positive or negative correlations with the efficacy of immunotherapeutic interventions. Furthermore, the augmentation of positively correlated “beneficial bacteria” is known to potentiate the therapeutic benefits of immunotherapy, thereby engendering tumor control and the amelioration of immune-related adverse reactions. Previous studies have already established the significant association between gut microbiota diversity and the outcomes of immunotherapy in lung cancer patients. The present study extends these findings by revealing that the Chao-1 index, Simpson index, and Shannon index in the R group surpassed those in the NR group. Notably, post-treatment analyses of selected Alpha diversity indices demonstrated an upward trajectory, indicating that the R group exhibited heightened richness and species diversity within its microbiota as compared to the NR group. Importantly, the findings underscore the substantial influence exerted by ICIs treatment on the richness and diversity of the microbiota in lung cancer patients (*p > 0.*05).

An increasing body of research has illuminated the profound influence wielded by the gut microbiota over the anti-tumor immune response via its regulatory impact on both the innate and adaptive immune systems. This phenomenon extends to the modulation and enhancement of the ICIs treatment response. Our experimental investigations unveiled the presence of unidentified cocci in lung cancer patients, accounting for approximately 1.2% of the relative abundance. Notably, there were marked shifts in relative abundance discernible before and after ICIs treatment, as well as within stratifications representing sustained benefits. The underlying causal mechanisms for these fluctuations remain to be elucidated. Importantly, we have incorporated these microbial strains into our genetic database for further analysis of the observed changes and their determinants. It is worth acknowledging that the catalogue of gut bacteria that are intricately associated with immunotherapy outcomes remains far from exhaustive. Moreover, the prevalence of putative taxa that discriminate between responders and non-responders frequently engenders contradictory findings across different cohort studies, even when focused on the same cancer type. Patients afflicted with advanced lung cancer invariably exhibit an immunosuppressed state, characterized by reduced immune cell activity, functional impairment, and immune evasion mechanisms. Within the intricate immune milieu of tumor microenvironments, a myriad of immune responses come into play. These encompass positive regulatory immune responses, notably CD3^+^ and CD4^+^ expressed in T helper cells (Th), and negative regulatory immune responses, including inhibitory T cells (Ts), T regulatory cells (TreGs), and myeloid suppressor cells. Tregs assume a pivotal role in restraining immune responses, thus promoting the immune escape mechanisms underlying tumor immune tolerance (Whiteside, 2018). Concurrently, myeloid suppressor cell subsets constitute critical contributors to tumor invasiveness, angiogenesis, metastasis propagation, while dampening the host’s anti-tumor immunity (Su et al., 2021). The fluctuations in the abundance of these immune cell subsets underscore the dynamics of the immune milieu. The CD4^+^/CD8^+^ ratio serves as an efficacious indicator for assessing immune functionality. Its elevation signifies an enhancement of immune functionality, while a decrease reflects a state of immune suppression (Ren, 2022). This study aims to explore the mechanism of immune checkpoint inhibitors and Chinese medicine in the treatment of lung cancer by investigating the relationship between intestinal microbes and the immune microenvironment. Such exploration is pivotal for enhancing the efficacy of lung cancer treatment. However, certain challenges were encountered during the study. The inclusion of patients undergoing immunocombined chemotherapy, as well as those receiving individual single-drug immunotherapy and combined local therapy, resulted in baseline instability and introduced some selection bias.

In recent years, Chinese medicine has made notable research advancements in the field of lung cancer immunotherapy. This holds significant research value in augmenting the immunity and anti-tumor effects of cancer patients through the regulation of intestinal flora. The intestinal microbiota serves as a key exogenous regulator influencing the tumor response to immunotherapy, and strategies to modulate intestinal bacteria for increased anti-cancer efficacy remain a common focus among researchers. Additionally, an increasing number of studies have affirmed the close relationship between traditional Chinese medicine and intestinal bacteria. However, there is limited research on the interplay between traditional Chinese medicine, intestinal microecology, and the immune microenvironment in lung cancer, necessitating further exploration.Furthermore, although it is established that immune checkpoint inhibitors therapy in lung cancer patients is linked to a favorable gut microbiota, the precise mechanism of interaction remains incompletely understood and validated. Moreover, the characteristic intestinal bacteria identified in various lung cancer experiments may differ, or there may be a multitude of them. The task of screening out beneficial bacteria and elucidating the synergistic interaction between these bacteria requires further investigation.

## Data availability statement

The original contributions presented in the study are publicly available. This data can be found at NCBI under BioProject ID PRJNA1063715.

## Ethics statement

The studies were conducted in accordance with the local legislation and institutional requirements. The participants provided their written informed consent to participate in this study.

## Author contributions

LL: Writing – original draft. HZ: Writing – original draft, Data curation. YW: Writing – original draft. ZP: Software, Writing – original draft. SX: Investigation, Writing – original draft. SL: Data curation, Writing – original draft. GZ: Data curation, Writing – original draft. WZ: Methodology, Writing – original draft. JL: Methodology, Writing – original draft. LH: Conceptualization, Investigation, Writing – original draft.
