# Supplementary material for: Exploring the relationship between intestinal microbiota and immune checkpoint inhibitors in the treatment of non-small cell lung cancer: insights from the “lung and large intestine stand in exterior-interior relationship” theory
Source: Front Cell Infect Microbiol. 2024 Feb 9;14:1341032. doi: 10.3389/fcimb.2024.1341032 (PMC10898591; doi:10.3389/fcimb.2024.1341032)
Supplement: Supplementary file 1 [file DataSheet_1.docx]

**Supplementary materials**

Table 1. Comparison of TCM syndrom changes before and after treatment (%)

| **TCM syndrome** | **Pre-treatment** | **Post-treatment** |
| --- | --- | --- |
| Syndrome of phlegm-damp obstructing lung | 3 (7.69) | 1 (2.56) |
| Syndrome of phlegm-heat obstructing lung | 3 (7.69) | 1 (2.56) |
| Syndrome of Qi stagnation and phlegm obstruction | 1 (2.56) | 1 (2.56) |
| Syndrome of phlegm and blood stasis | 2 (5.13) | 1 (2.56) |
| Syndrome of spleen-lung Qi deficiency | 19 (48.71) | 15 (33.33) |
| Syndrome of Qi-Yin deficiency | 11 (28.20) | 8 (20.51) |

Table 2. Changes of TCM syndromes for the R group and NR group (%)

| **Group** | **Case** | **Spleen-lung Qi deficiency syndrome** | | **Qi-Yin deficiency syndrome** | |
| --- | --- | --- | --- | --- | --- |
|  |  | **Pre-treatment** | **Post-treatment** | **Pre-treatment** | **Post-treatment** |
| R Group | 24 | 12 (50.00) | 8 (33.30) | 7 (29.17) | 5 (20.83) |
| NR Group | 15 | 7 (46.67) | 5 (33.33) | 4 (26.67) | 3 (20.00) |
| $X^{2}$ |  | 0.041 | 0.000 | 0.028 | 0.004 |
| *P* |  | 0.839 | 1.000 | 0.866 | 0.950 |

Table 3. Species differences between BT group and AT group - biomarker

| **Hierarchy** | **Strain** | **Average abundance %** | | ***p*-value** |
| --- | --- | --- | --- | --- |
|  |  | **AT** | **BT** |  |
| **Family** | Pseudoalteromonadaceae | 0.1 | 0.5 | 0.042 |
|  | Alcaligenaceae | 0.1 | 0.3 | 0.046 |
|  | Neisseriaceae | 0.03 | 0.1 | 0.042 |
| **Genus** | Roseburia | 0.7 | 1.97 | 0.011 |
|  | Pseudoalteromonas | 0.1 | 0.48 | 0.042 |
|  | Dorea | 0.26 | 0.52 | 0.024 |
|  | Lachnospiraceae | 0.22 | 0.45 | 0.025 |
|  | Castellaniella | 0.06 | 0.21 | 0.039 |
|  | Coprococcus | 0.26 | 0.44 | 0.029 |
| **Species** | Roseburia_inulinivorans | 0.53 | 1.60 | 0.023 |
|  | Pseudoalteromonas_  luteoviolacea | 0.10 | 0.48 | 0.042 |
|  | Parabacteroides_merdae | 1.15 | 0.69 | 0.040 |
|  | Lachnospiraceae | 0.16 | 0.38 | 0.020 |
|  | Roseburia_intestinalis | 0.17 | 0.36 | 0.030 |
|  | Streptococcus | 0.03 | 0.13 | 0.042 |

**Table 4a Changes in relative abundance of family, genus and species before and after ICIs treatment**

| **Hierarchy** | Enrichment reduction | Increase in  abundance | Disappear |
| --- | --- | --- | --- |
| Family | Pseudoalteromonadaceae |  | Micropepsaceae |
|  | Alcaligenaceae |  |  |
|  | Neisseriaceae | There is no. | Parvibaculaceae |
|  | Moraxellaceae |  |  |
|  | Idiomarinaceae |  | Methylococcaceae |
|  | Inquilinaceae |  |  |
| Genus | Roseburia | Dielma | Psychrobacter |
|  | Pseudoalteromonas |  |  |
|  | Dorea |  | Marinospirillum |
|  | Lachnospiraceae_NK4A136_group | Gardnerella |  |
|  | Castellaniella |  | Micropepsis |
|  | Coprococcus |  |  |
|  | Acinetobacter | DEV008 | Rhodovastum |
|  | Cupriavidus |  |  |
|  | Inquilinus | Tepidiphilus | Parvibaculum |
|  | Aliidiomarina |  |  |
|  | Flaviflexus |  |  |
| Speciecs | Roseburia_inulinivorans | Acinetobacter | Psychrobacter_alimentarius |
|  | Parabacteroides_merdae |  |  |
|  | Dorea_formicigenerans |  |  |
|  | Pseudoalteromonas_luteoviolacea |  |  |
|  | Lachnospiraceae_bacterium_GAM79 |  | Marinospirillum_minutulum |
|  | Stenotrophomonas_acidaminiphila |  |  |
|  | Roseburia_intestinalis |  |  |
|  | Streptococcus_sp_FF10 |  |  |
|  | Dorea_formicigenerans |  |  |

**Table 4b接受治疗的R组和NR组在目、科、属种上的组间差异**

| **Hierarchy** | The concentration of group R is high | It is only enriched in group R | The concentration of NR group was high |
| --- | --- | --- | --- |
| Order | Monoglobales |  |  |
|  | Catenulisporales | There is no. | Acetobacterales |
|  | Peptococcales |  |  |
|  | Cardiobacteriales |  |  |
| Family | Butyricicoccaceae | Parachlamydiaceae | Catenulisporaceae |
|  | Intrasporangiaceae |  |  |
|  | Monoglobaceae |  |  |
|  | Sphingobacteriaceae |  | Spirosomaceae |
|  | Actinospicaceae | unidentified_Clostridia_UCG-014 |  |
|  | unidentified_IMCC26256 |  |  |
|  | Frankiaceae |  | Acetobacteraceae |
|  | Peptococcaceae |  |  |
|  | Cardiobacteriaceae |  |  |
| Genus | Sinomonas | Pseudarcicella | Terracidiphilus |
|  | Burkholderia-Caballeronia-Paraburkholderia |  |  |
|  | Butyricicoccus | Companilactobacillus |  |
|  | Dyella |  |  |
|  | Monoglobus |  | Catenulispora |
|  | Mucilaginibacter | unidentified_Clostridia_UCG-014 |  |
|  | Oryzihumus |  |  |
|  | Granulicella |  |  |
|  | Herbaspirillum |  | GCA-900066755 |
|  | Terrabacter |  |  |
|  | Tyzzerella |  |  |
|  | Actinospica |  |  |
|  | Edaphobacter | Actinocatenispora | Lactococcus |
|  | unidentified_IMCC26256 |  |  |
|  | Jatrophihabitans |  |  |
|  | Cardiobacterium |  |  |
| Species | Terrabacter_sp | Clostridiales_bacterium_oral_taxon_075 | Blautia_hydrogenotrophica |
|  | Sinomonas_atrocyanea |  |  |
|  | Intestinimonas_butyriciproducens |  |  |
|  | Streptococcus_anginosus |  |  |
|  | Cardiobacterium_valvaru | Faecalitalea_cylindroides |  |
|  | Clostridium_scindens |  |  |
|  | Persicaria_minor | Pseudarcicella_hirudinis |  |
|  | Lactococcus_garvieae |  |  |
|  | Ruminococcaceae_bacterium_GD6 |  |  |

Table 5. Comparison of peripheral immune parameters before and after ICIs treatment (X±S)

| **Index** | **BT Set** | **AT Group** | **T-value** | ***p*-value** |
| --- | --- | --- | --- | --- |
| CD3^+^(%) | 67.99 + / - 9.617 | 74.36 + / - 10.45 | 3.363 | 0.002 |
| CD4^+^(%) | 34.13 + / - 10.70 | 39.33 + / - 6.34 | 2.856 | 0.008 |
| CD8^+^(%) | 31.77 + / - 12.09 | 27.46 + / - 6.39 | 2.084 | 0.046 |
| CD4^+^/CD8(%) | 1.32 + / - 0.75 | 1.58 + / - 0.40 | 2.491 | 0.019 |
| Treg/CD4^+^ | 5.46 + / - 2.07 | 4.60 + / - 0.77 | 2.395 | 0.023 |
| CD3^+^ | 732.05 + / - 413.21 | 621.61 + / - 356.99 | 1.815 | 0.080 |
| CD4^+^ | 380.91 + / - 248.74 | 321.43 + / - 180.76 | 1.628 | 0.114 |
| CD8^+^ | 344.90 + / - 216.08 | 211.84 + / - 127.31 | 3.658 | 0.001 |
| M-MDSC/MDSC | 4.30 + / - 4.87 | 2.70 + / - 5.56 | 1.501 | 0.144 |
| G-MDSC/MDSC | 0.81 + / - 1.52 | 0.51 + / - 0.98 | 1.758 | 0.089 |

Table 6. Comparison of immune parameters in patients with different therapeutic effects before treatment (X±S)

| **Index** | **Pre-treatment** | | **T-value** | ***p*-value** |
| --- | --- | --- | --- | --- |
|  | **NR (n=10)** | **R (n=20)** |  |  |
| CD3^+^(%) | 67.39 + / - 8.23 | 68.29 + / - 10.43 | 0.238 | 0.814 |
| CD4^+^(%) | 33.60 + / - 9.18 | 34.40 + / - 11.60 | 0.190 | 0.851 |
| CD8^+^(%) | 31.30 + / - 14.37 | 32.00 + / - 11.19 | 0.147 | 0.884 |
| CD4^+^/CD8^+^(%) | 1.42 + / - 0.93 | 1.27 + / - 0.66 | 0.521 | 0.607 |
| CD3^+^ | 741.40 + / - 403.20 | 727.38 + / - 428.41 | 0.086 | 0.932 |
| CD4^+^ | 387.46 + / - 249.89 | 377.63 + / - 254.61 | 0.100 | 0.921 |
| CD8^+^ | 346.77 + / - 237.16 | 343.97 + / - 211.22 | 0.033 | 0.974 |
| Treg/CD4^+^ | 4.96 + / - 2.82 | 5.71 + / - 1.61 | 0.938 | 0.356 |
| M-MDSC/MDSC | 4.71 + / - 5.17 | 4.10 + / - 4.85 | 0.318 | 0.753 |
| G-MDSC/MDSC | 1.250 + / - 2.25 | 0.58 + / - 0.99 | 1.138 | 0.2265 |

Table 7. Comparison of immune parameters between spleen-lung Qi deficiency syndrome and Qi-Yin deficiency syndrome (X±S)

| **Index** | **Pre-treatment** | | **T-value** | ***p-*value** |
| --- | --- | --- | --- | --- |
|  | **Spleen-lung Qi deficiency syndrome (n=19)** | **Qi-Yin deficiency syndrome (n=11)** |  |  |
| CD3^+^ (%) | 66.71 + / - 9.47 | 70.20 + / - 9.91 | 0.959 | 0.347 |
| CD4^+^ (%) | 34.55 + / - 10.69 | 33.42 + / - 11.20 | 0.274 | 0.786 |
| CD8^+^ (%) | 30.50 + / - 13.92 | 39.96 + / - 8.20 | 0.748 | 0.461 |
| CD4^+^/CD8^+^(%) | 1.45 + / - 0.79 | 1.10 + / - 0.63 | 1.214 | 0.235 |
| CD3^+^ | 762.18 + / - 482.36 | 680.00 + / - 267.65 | 0.518 | 0.608 |
| CD4^+^ | 406.43 + / - 288.68 | 336.81 + / - 161.39 | 0.733 | 0.470 |
| CD8^+^ | 330.65 + / - 229.95 | 314.45 + / - 136.52 | 0.212 | 0.834 |
| Treg/CD4^+^ | 5.26 + / - 2.28 | 5.80 + / - 1.69 | 0.684 | 0.500 |
| M-MDSC/MDSC | 5.05 + / - 5.43 | 3.00 + / - 3.59 | 1.122 | 0.271 |
| G-MDSC/MDSC | 1.02 + / - 1.82 | 0.44 + / - 0.71 | 0.999 | 0.372 |
